# Complete coding sequence of *Rift Valley fever virus* identified by metagenomic sequencing in patient with undifferentiated febrile illness at Marigat sub-district hospital, Kenya

**DOI:** 10.1128/mra.00366-24

**Published:** 2024-07-31

**Authors:** Allan Lemtudo, Gathii Kimita, George Awinda, Beth Mutai, John Waitumbi

**Affiliations:** 1 Kenya Medical Research Institute/Walter Reed Army Institute of Research—Africa/Basic Science Laboratory, Kisumu, Kenya; DOE Joint Genome Institute, Berkeley, California, USA

**Keywords:** *Rift Valley fever virus*, metagenomic sequencing, febrile illness, disease outbreaks, mosquito borne

## Abstract

We report on the complete coding sequence of *Rift Valley Fever Virus* inadvertently identified through metagenomics in a child with undifferentiated fever at Marigat sub-county hospital, Kenya. On phylogeny, the genome clustered with sequences obtained during the 2017 human outbreak in Uganda and the 2021 cattle outbreak in Kiambu, Kenya.

## ANNOUNCEMENT

*Rift Valley fever virus* (*RVFV*) is a *Phlebovirus* in the *Bunyaviridae* family that is transmitted by mosquitoes that largely affect livestock, but can also infect humans (1, 2). Outbreaks occur after heavy rains that create mosquito breeding grounds (2, 3). In 2018, conditions in Wajir and Marsabit counties, Kenya, led to RVF cases (4). In December 2019, a blood sample that had been obtained in August 2018 from a febrile child at Marigat sub-county hospital (648 km from the nearest county) had short genomic sequences that mapped to *RVFV*. Presence of *RVFV* in the sample was confirmed by RT-qPCR (Altona Diagnostics GmbH, Hamburg Germany) in 7500 fast machine (Applied Biosystem, CA, USA). Extended screening comprising 44 other samples collected from febrile patients around the same time as the index case (Table 1), revealed two more *RVFV*-positive samples (Cts 19.3 and 34.3).

**TABLE 1:** Demographic and clinical data showing age, gender, risk exposure (contact with animals), and reported symptoms among 45 patients who were tested for RVFV^*a*^

Variable	Category	Frequency	(%)
Demographic distribution			
Sex	Male	20	44.4
	Female*	25	55.6
Age (years)	≤5	10	22.2
	06-Dec*	19	42.2
	13–19	11	24.4
	20–35	4	8.9
	≥35	1	2.2
Risk exposure			
Contact with cows		26*	57.8
Contact with goats		32	71.1
Contact with sheep		23*	51.1
Symptoms			
Fever (≥38⁰)		44*	100
Headache		44*	100
Joint aches		37*	82.2
Chills		34*	75.6
Muscle aches		33*	73.3
Vomiting		17	37.8

^*a*^ The asterisks indicate demographic details associated with index case that spurred investigation for *RVFV*.

RNA was isolated from serum of positive samples using the MagMAX Pathogen RNA/DNA Kit (Applied Biosystems), then depleted of host genomic DNA with TURBO DNase Kit (Invitrogen, CA, USA), followed by first-strand cDNA synthesis with Superscript IV RT kit (Invitrogen) using SISPA primers (5). The second strand was synthesized by a Klenow reaction (New England Biolabs, MA, USA), followed by random fragment amplification with MyTaq Red master mix (Meridian Bioscience Inc, OH, USA). Amplicons were used to prepare sequence libraries using Collibri ES DNA library prep kit (Invitrogen, CA, USA) and paired ends were sequenced using v3 chemistry on a Miseq (Illumina, CA, USA). The ngs_mapper pipeline v1.4.2 (https://github.com/VDBWRAIR/ngs_mapper) was used to quality filter, map, and generate consensus sequences, which were curated and annotated in Geneious Prime v2023.2.1 using a customized database of *RVFV* genomes derived from NCBI Genbank. Lineage assignment was conducted using rvfvtyping v1.0 (6). Only the study sample with a Ct value of 19.3 yielded useable sequences and was analyzed for phylogenetic assignment in MAFFT v7 (7) together with sequences obtained from the BV-BRC database. Nucleotide substitution models were tested in JModelTest v2 (8), and the GTR + GI substitution model was used to infer a maximum likelihood phylogeny with PhyML v3 (9).

**Fig 1:**
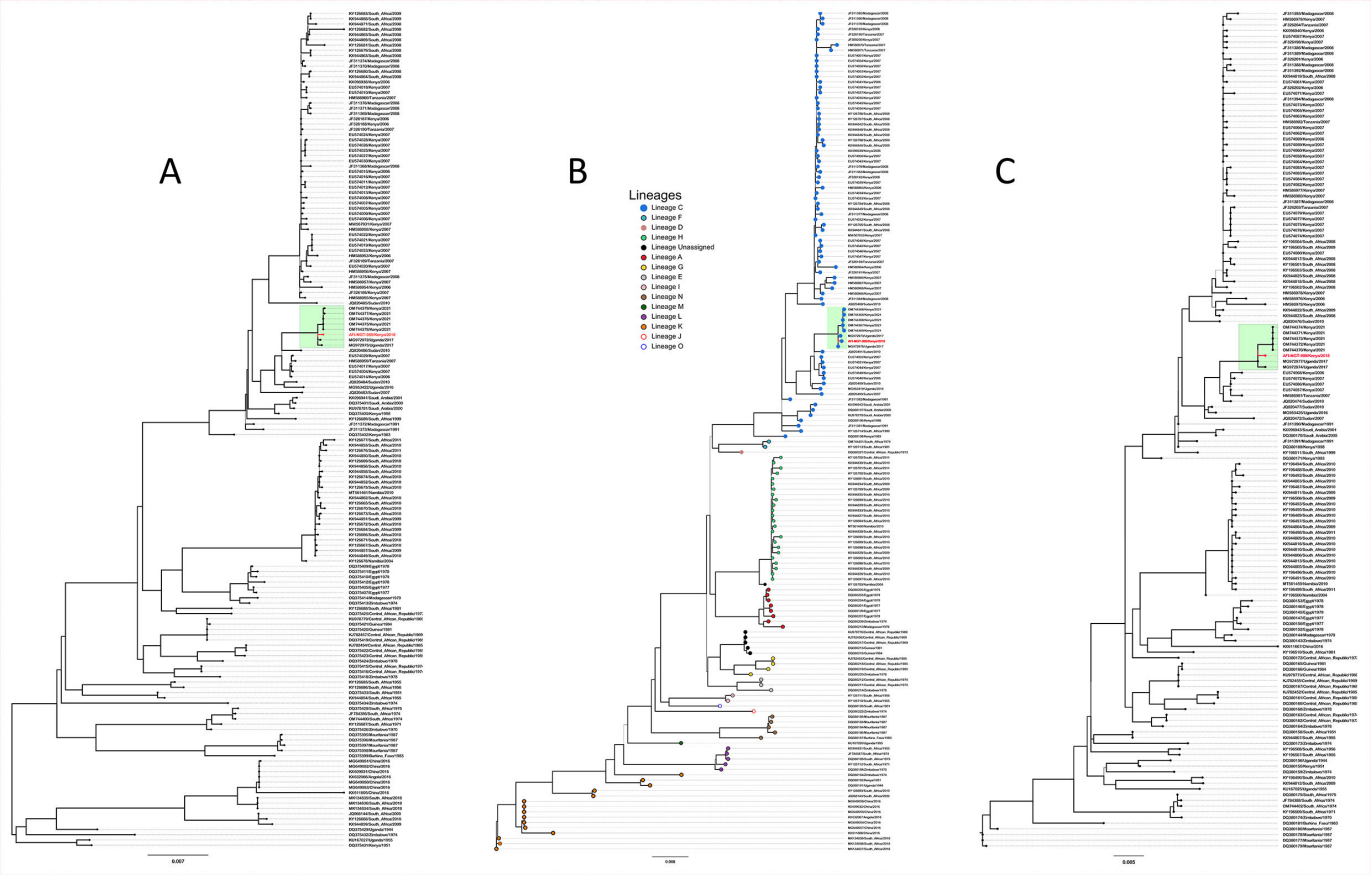
Phylogenetic tree drawn from 477 RVFV L (Panel A), M (Panel B), and S (Panel C) genomes from BV-BRC (Bacterial and Viral Bioinformatics Resource Center). The study genome (indicated with red fonts) branched with lineage C genomes and clustered with the 2017 human RVFV outbreak in Uganda and the 2021 cattle outbreak in Kenya (highlighted in green).

The study genome yielded 1,611,904 reads that mapped to a 12-kilobase *RVFV* reference genome: NC_014397 corresponding to the L segment (6,397 bp, 43.4% GC, mean coverage 34,331×); NC_014396 to M segment (3,875 bp, 46.3% GC, mean coverage 33,671×), and NC_014395 to S (1,684 bp, 51.1% GC, mean coverage 22,465×). The study genome was classified as lineage C (Fig. 1, red fonts), and clustered in a clade comprising sequences from the 2017 human *RVFV* outbreak in Uganda (10) and the 2021 cattle outbreak in Kenya (6) (Fig. 1, highlighted in green).

We detected *RVFV* in febrile patients without travel history to outbreak-affected counties, suggesting exposure through transported animals, meat, or milk. Enzootic *RVFV* circulation in Baringo County is also possible due to its high-risk status (11). The C lineage, previously linked to major RVF outbreaks in East Africa (including the 2006–2007 outbreak in Kenya, Tanzania, and Somalia), may have reservoirs in the country (12).

## Data Availability

The genome sequences reported in this work are available in Genbank under the following accession numbers: OR972326–OR972328. The raw sequencing reads are available in NCBI’s Sequence Read Archive (SRA) under BioProject accession number PRJNA1099136 and SRA Run ID. SRR28624259.
